# Early Clinical Experience with the Novel A-Stream Glaucoma Shunt Combined with Mid-Posterior Tenon’s Capsule Advancement Flap

**DOI:** 10.3390/jcm15010056

**Published:** 2025-12-21

**Authors:** Young Hoon Hwang, Sharon Lee, Mijin Kim, Jaewan Choi

**Affiliations:** Central Seoul Eye Center, Seoul 04427, Republic of Korea; brainh@hanmail.net (Y.H.H.); lsr6882@nate.com (S.L.); happypinnk52@gmail.com (M.K.)

**Keywords:** glaucoma, surgery, intraocular pressure

## Abstract

**Objectives:** The aim of this study was to evaluate the clinical outcomes and surgical techniques of A-stream Glaucoma Shunt (A-stream; MICROT Inc.) implantation. **Methods:** A retrospective review of 59 eyes from 59 patients who underwent A-stream implantation was conducted. Eyes were divided into two groups: group A (25 eyes), where the distal tip of the ripcord was left exposed and the device was covered with conjunctiva, scleral flap, pericardium patch graft, or mid-posterior Tenon’s capsule advancement flap (MPTAF); and group B (34 eyes), where the ripcord was shaped into a loop at the corneal limbus and the device was covered with MPTAF. Intraocular pressure (IOP), number of IOP-lowering medications, visual acuity, and postoperative complications were analyzed. Success was defined as an IOP ≤ 21 mmHg with ≥20% reduction from baseline, without clinically significant hypotony, and no reoperation for glaucoma. **Results:** At 6 months, the mean IOP decreased significantly in both groups (group A: 27.4 ± 9.8 mmHg to 15.2 ± 5.1 mmHg; group B: 25.1 ± 11.6 mmHg to 14.0 ± 7.0 mmHg, *p* < 0.001). Success rates were 76.0% in group A and 88.2% in group B (*p* = 0.207). Early postoperative hypotony occurred in 8.0% of group A and 14.7% of group B, and these cases resolved without further intervention within 1 month. Ripcord complications were observed in 28.0% of group A, but none in group B. **Conclusions:** A-stream implantation with MPTAF and a limbal ripcord loop is an effective and safe procedure with minimal risk of device exposure and ripcord-related complications.

## 1. Introduction

A-stream Glaucoma Shunt (A-stream; MICROT Inc., Seoul, Republic of Korea) is a recently introduced minimally invasive glaucoma drainage device (GDD) designed to divert aqueous humor from the anterior chamber to the post-limbal sub-Tenon’s space [1]. The device consists of a 6 mm tube with an internal diameter of 100 µm that serves as the pathway for aqueous outflow, a wing attached to the tube to prevent anterior migration, and a pre-placed 7-0 nylon intraluminal thread that limits excessive aqueous drainage [1]. In addition, this intraluminal thread serves another role by enabling stepwise postoperative flow control through partial or complete removal, and is therefore commonly referred to as a “ripcord.” The concept of a tube with a pre-placed removable intraluminal ripcord has been applied either to membrane–tube type GDDs [2]. Previous laboratory and clinical studies using a tube with a profile similar to that of A-stream have demonstrated stable and predictable aqueous drainage [2,3].

To date, the only formally reported technique of A-stream implantation involves conjunctival incision, scleral flap formation, tube insertion under the scleral flap, and placement of the extraocular portion of the ripcord through the conjunctival surface over the bleb [1]. We considered that, while maintaining the fundamental concept of A-stream implantation, alternative surgical approaches could be applied. In this study, we sought to investigate such options, particularly with respect to device coverage and ripcord placement.

## 2. Materials and Methods

### 2.1. Participants

This retrospective, observational study protocol was approved by the Institutional Review Board at Korea National Institute for Bioethics Policy, Republic of Korea. Informed consent was waived due to the retrospective study design. All procedures conformed to the guidelines of the Declaration of Helsinki. In this report, we enrolled 59 eyes from 59 patients with glaucoma who underwent A-stream implantation at the Central Seoul Eye Center, Seoul, Republic of Korea, between June 2024 and October 2024, with a follow-up period of more than six months.

Each participant underwent a comprehensive ophthalmic examination that included visual acuity assessments, intraocular pressure (IOP) measurements using a Goldmann applanation tonometer, anterior segment examination using slit-lamp biomicroscopy, and optic nerve head and fundus examination. Glaucoma was defined as the presence of glaucomatous changes (i.e., a large cup-to-disc ratio and neuroretinal rim narrowing) in the optic nerve head and a retinal nerve fiber layer defect observed in fundus examination. Automated visual field testing was performed using a Humphrey Visual Field Analyzer (Carl Zeiss Meditec, Dublin, CA, USA) with the 24-2 Swedish Interactive Thresholding Algorithm standard strategy.

The indication for A-stream implantation was medically uncontrolled glaucoma in patients aged over 18 years, regardless of glaucoma severity and type, visual acuity, or previous history of ocular surgery. In eyes with a history of glaucoma surgery, A-stream implantation was considered when the conjunctival condition was deemed adequate for the formation of a functional filtering bleb.

### 2.2. Surgical Technique

In general, we followed the basic principles of the A-stream implantation procedure, which included local anesthesia, fornix-based conjunctival incision, application of a 0.04% mitomycin C-soaked sponge for three to five minutes, balanced salt solution irrigation, creation of the tube entry site using a needle, implantation of the A-stream into the anterior chamber with fixation to the scleral surface using 10-0 nylon sutures, positioning of the distal tip of A-stream in the subconjunctival space, placement of a ripcord for postoperative on-demand removal, and conjunctival closure [1].

**Figure 1:**
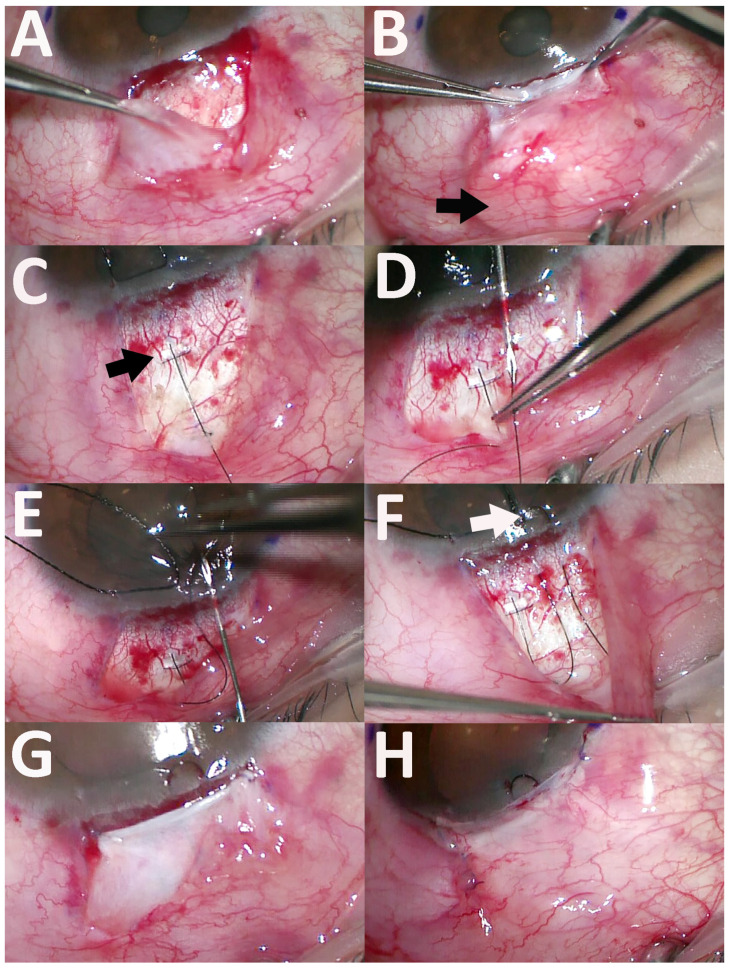
Surgical techniques of the A-stream implantation combined with mid-posterior Tenon’s capsule advancement flap (MPTAF) and the use of an anterior limbal ripcord loop. (**A**) Following a fornix-based conjunctival incision, forceps were inserted beneath the conjunctiva, and the anterior border of the mid-posterior Tenon’s capsule—typically located near the rectus muscle insertions—was gently pulled forward to create the MPTAF. (**B**) Antimetabolite application was performed by placing a mitomycin C-soaked sponge (arrow) beneath the MPTAF. (**C**) The tube was inserted 1 mm posterior to the limbus directly through the sclera. The wing (arrow) was positioned at the tube entry site to inhibit anterior migration, and a 10-0 nylon fixation suture was placed just posterior to the wing. (**D**) A bent 30-gauge needle was inserted from anterior to posterior at the corneal limbus to create a tunnel. The ripcord was then inserted into the lumen of the needle and pulled out anteriorly through the corneoscleral tunnel. (**E**) A second shallow passage was created lateral to the first tunnel, this time from posterior to anterior, and the ripcord was retrieved posteriorly. (**F**) This formed an anterior limbal loop (arrow). (**G**) The anterior margins of the MPTAF were anchored to the scleral surface. (**H**) The conjunctiva was closed with sutures.

To expand our approach beyond the previously suggested methods, we explored alternative surgical strategies, particularly regarding device coverage and ripcord placement techniques. For device coverage, various methods can be employed, including scleral flap formation as originally suggested by Park et al. [1], conjunctival coverage alone, pericardial patch grafting, or the use of preexisting Tenon’s capsule. When a scleral flap was created, the tube entry site was established at the limbus beneath the flap using a 30-gauge needle, and the device was secured with 10-0 nylon sutures placed both anterior and posterior to the wing. In cases without a scleral flap, the tube was inserted 1 mm posterior to the limbus, directly through the sclera, using a 27-gauge needle (Figure 1, Video S1). Using this technique, the wing was positioned at the tube entry site to inhibit anterior migration, and a 10-0 nylon fixation suture was placed just posterior to the wing to prevent posterior migration of the A-stream.

### 2.3. Mid-Posterior Tenon’s Capsule Advancement Flap Technique

The importance of Tenon’s capsule coverage at the operated site has been emphasized in trabeculectomy [4,5] and in the management of limbal or scleral ischemia [6]. Tenon’s capsule envelops the eyeball from the corneal limbus to the optic nerve and can be divided into anterior and mid-posterior portions [7]. According to histological analysis, the mid-posterior Tenon’s capsule begins where the rectus muscles insert into the capsule and exhibits greater thickness compared to the anterior Tenon’s capsule [7]. In addition, biomechanical testing demonstrated that the mid-posterior Tenon’s capsule withstood greater peak forces during suture pull-out tests than the anterior portion [7]. Therefore, we hypothesized that advancement of the mid-posterior Tenon’s capsule could serve as a useful option for preventing postoperative A-stream exposure. When applying the MPTAF technique, the following key points were carefully observed.

1. Advancement of the mid-posterior Tenon’s capsule was initiated by inserting fine-tooth forceps beneath the conjunctiva following a fornix-based incision and gently pulling forward the anterior border of the mid-posterior Tenon’s capsule, typically located near the insertion of the rectus muscles. After mobilizing an initial portion of the capsule, the adjacent tissue was progressively advanced until a sufficient area was available to cover the A-stream without traction. This maneuver created the mid-posterior Tenon’s capsule advancement flap (MPTAF; Figure 1, Video S1).
2. Application of antimetabolite was performed by placing a mitomycin C-soaked sponge beneath the MPTAF. Care was taken to ensure that the sponge did not come into contact with the overlying conjunctiva, minimizing the risk of epithelial toxicity or delayed healing (Figure 1, Video S1).
3. Fixation of the MPTAF was achieved by anchoring both anterior margins of the flap to the underlying scleral surface just posterior to the limbus prior to conjunctival closure, ensuring stable coverage of the A-stream implant (Figure 1, Video S1).

### 2.4. Ripcord Placement

For the ripcord placement, two techniques were employed. In the posterior placement method, the distal end of the ripcord was either left completely or partially exposed outside the conjunctiva posterior to the A-stream, similar to the method described by Park et al. [1]. In the posterior placement technique, the ripcord was positioned beneath the conjunctiva along the scleral surface. Its distal end was externalized by inserting a bent 30-gauge needle from outside the conjunctiva toward the scleral surface and passing the ripcord through the needle lumen, allowing both the needle and ripcord to be withdrawn together. The exposed portion was secured to the conjunctival surface by one of the following methods: suturing with 10-0 nylon, creating a knot to prevent retraction, or making an additional passage through the adjacent conjunctiva.

The anterior limbal loop technique involved creating a loop at the corneal limbus and securing it via partial sub-scleral passes to reduce mobility and minimize irritation. To create an anterior loop of the ripcord, a bent 30-gauge needle was first inserted from anterior to posterior at the corneal limbus, approximately 2–3 mm away from the tube entry site (Figure 1, Video S1). The needle was advanced in a shallow trajectory beneath the surface of the cornea and sclera, creating a tunnel of approximately 2–3 mm in length. The ripcord was then inserted into the lumen of the needle and pulled out anteriorly through the tunnel. To complete the loop, a second shallow passage was created from posterior to anterior, approximately 1–2 mm lateral to the first tunnel. The ripcord was inserted into the lumen of the needle and pulled posteriorly through the second tunnel, thereby forming a loop across the two sub-scleral tracks (Figure 1, Video S1).

Postoperatively, levofloxacin eye drops were administered four times daily, and prednisolone acetate 1% eye drops were instilled 6 to 8 times daily during the first 1–2 weeks. Thereafter, steroid drops were gradually tapered depending on each eye’s condition.

For ripcord removal, we applied criteria similar to those suggested by Park et al. [1]. In general, removal was considered when IOP exceeded targeted level or when bleb morphology suggested a failing bleb, such as the presence of encapsulation or increased vascularization.

### 2.5. Outcome Measures

IOP, number of IOP-lowering medications, best-corrected visual acuity (BCVA) expressed as logarithm of the minimum angle of resolution (logMAR), corneal endothelial cell count (ECC), and complications were assessed at baseline and at 1 week, 1 month, 3 months, and 6 months thereafter.

To evaluate the utility of various surgical techniques, the eyes were divided into two groups. In the initial 25 eyes (group A), which underwent mixed techniques, the distal tip of the ripcord was left exposed outside the conjunctiva, and the device was covered using one of several methods: conjunctiva alone, scleral flap, pericardial patch graft, or MPTAF. In the subsequent 34 eyes (group B), which received a uniform technique, the ripcord was fashioned into a loop at the corneal limbus, and the device was covered exclusively with MPTAF.

In the present report, the definition of success was adopted from a previous study to maintain consistency [1]. Success was defined as an IOP ≤ 21 mmHg with a ≥ 20% reduction from baseline without clinically significant hypotony (IOP < 6 mmHg persisting >1 month or with hypotony maculopathy), with (qualified success) and without (complete success) the use of IOP-lowering medications. Hypotony can be further classified into two types: numeric hypotony, defined as an IOP < 6 mmHg, and symptomatic hypotony, characterized by an IOP < 6 mmHg accompanied by signs such as shallow anterior chamber, choroidal effusion, or hypotony maculopathy [8]. Need for additional glaucoma surgery or the experience of vision-threatening complications—such as suprachoroidal hemorrhage, retinal detachment, endophthalmitis, corneal decompensation, or loss of light perception—was considered a failure. Control of IOP after ripcord removal or needling revision was not considered a failure. In this study, we defined ripcord dislocation as complete disengagement of the ripcord from the tube lumen, and ripcord retraction as unintended migration of the ripcord beneath the conjunctival surface.

### 2.6. Statistical Analyses

Changes in variables between baseline and follow-up visits were analyzed using the paired *t*-test, while differences between the two groups were assessed using the independent *t*-test, chi-square test, or Fisher’s exact test. Success rate was calculated using Kaplan–Meier survival analysis. Cox proportional hazards model was used to identify factors affecting survival. A *p*-value < 0.05 was considered statistically significant. Statistical analyses were performed using SPSS for Windows (version 24.0; IBM Corp., Armonk, NY, USA).

## 3. Results

A total of 59 eyes from 59 Korean patients (15 females and 44 males) were enrolled in the study. The clinical characteristics at baseline are presented in Table 1. The mean age was 58.6 ± 12.8 years. The most common diagnosis was primary open-angle glaucoma (66.1%), followed by pseudoexfoliation glaucoma (13.6%) and uveitic glaucoma (8.5%). At the baseline visit, the mean visual acuity, IOP, and number of IOP-lowering medications were 0.40 ± 0.73 logMAR, 26.1 ± 10.8 mmHg, and 2.5 ± 0.8, respectively. The mean deviation and visual field index from the visual field test were −16.0 ± 8.5 dB and 53.6 ± 28.5%, respectively. The number of eyes with a history of prior incisional glaucoma surgery, including trabeculectomy or GDD implantation was 7 (28.0%) in group A and 5 (14.7%) in group B (*p* = 0.177). The proportion of eyes with pseudophakia was similar between the two groups (*p* = 0.305), and all 3 eyes with angle-closure glaucoma in Group B were pseudophakic. There was no difference in clinical characteristics between the two groups, with exception of a greater number of IOP-lowering medications in group A (2.7 ± 0.8) compared to group B (2.2 ± 0.7, *p* = 0.011).

In Group A, the A-stream was covered with a scleral flap in 2 eyes, conjunctiva alone in 3 eyes, a pericardial patch graft in 5 eyes, and the MPTAF technique in 15 eyes, with the ripcord left exposed posterior to the device. In Group B, all eyes received MPTAF coverage, and the ripcord was positioned using the anterior limbal loop technique. MPTAF was feasible in all eyes, including those with thin or scarred conjunctiva and anterior Tenon’s capsule when the scarring was not severe.

**Figure 2:**
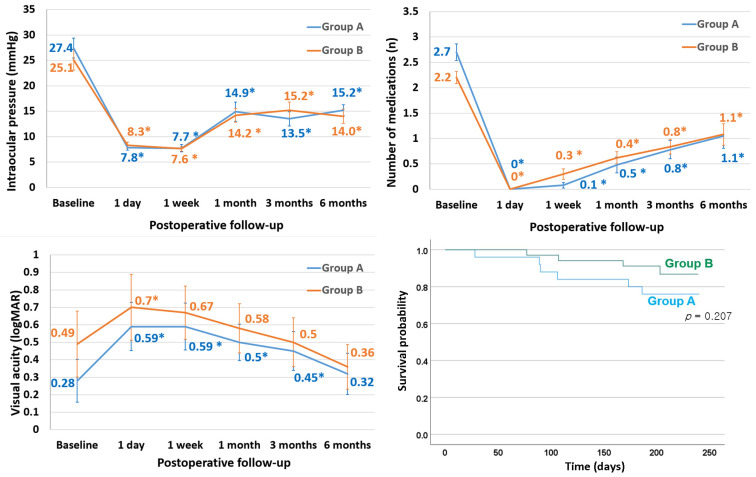
Changes in intraocular pressure (**top left**), number of medications (**top right**), visual acuity (**bottom left**), and the Kaplan–Meier survival curve for the cumulative qualified success rate (**bottom right**) following A-stream implantation. *: significant difference (*p* < 0.05) from baseline. logMAR, logarithm of the minimum angle of resolution.

Changes in IOP are shown in Figure 2. At 6 months postoperatively, the mean IOP decreased from 26.1 ± 10.8 mmHg to 14.5 ± 6.2 mmHg (*p* < 0.001). In group A, the mean IOP decreased from 27.4 ± 9.8 mmHg to 15.2 ± 5.1 mmHg (*p* < 0.001), while in group B, the mean IOP decreased from 25.1 ± 11.6 mmHg to 14.0 ± 7.0 mmHg (*p* < 0.001).

Changes in the number of IOP-lowering medications are also shown in Figure 2. At 6 months postoperatively, the average number of medications decreased from 2.5 ± 0.8 to 1.1 ± 1.1 (*p* < 0.001). In group A, the mean number decreased from 2.7 ± 0.8 to 1.1 ± 1.1 (*p* < 0.001), while in group B, it decreased from 2.2 ± 0.7 to 1.1 ± 1.1 (*p* < 0.001).

No significant change in BCVA was observed at 6 months postoperatively in either group (*p* > 0.05, Figure 2). In group A, a decrease in BCVA was observed on postoperative day 1, week 1, and at months 1 and 3, while in group B, a transient decrease in BCVA was noted on postoperative day 1 only(*p* < 0.05, Figure 2).

When the ECC was analyzed, no significant change was observed in either group at any visit (*p* > 0.05). The mean ECC was 2173.2 ± 645.9 cells/mm^2^ at the baseline visit and 2362.7 ± 577.1 cells/mm^2^ at the final visit.

Qualified success rates were observed in 86.4% of cases overall, with 76.0% for group A eyes and 88.2% for group B eyes (*p* = 0.207, Figure 2). Complete success was achieved in 80.0% of cases, with 72.0% for group A and 85.3% for group B (*p* = 0.316). Age, type of glaucoma, IOP level, number of IOP-lowering medications, and ripcord removal did not significantly affect the success rate (*p* > 0.05).

The most common complication was hypotony (Table 2). Numeric hypotony was observed in 11 of 25 eyes (44.0%) in group A and 13 of 34 eyes (38.2%) in group B at 1 week postoperatively (*p* = 0.429). At 1 month postoperatively, the incidence decreased to 2 eyes (8.0%) in group A and 1 eye (3.7%) in group B (*p* = 0.471). Symptomatic hypotony, defined as hypotony with choroidal effusion, was observed in 2 eyes (8.0%) in group A and 5 eyes (14.7%) in group B (*p* = 0.359). These symptoms resolved without further intervention in all affected eyes, and no cases of hypotony maculopathy were observed. Additionally, hyphema was noted in 1 eye of group A, which spontaneously resolved. Needling revision was performed in 4 eyes in Group A and 5 eyes in Group B.

Ripcord dislocation and retraction into the subconjunctival space were observed in 3 eyes (12.0%) and 4 eyes (16.0%) of group A eyes, respectively, whereas no ripcord-related complications occurred in group B. Among eyes with ripcord dislocation, numeric hypotony (IOP < 6 mmHg) was observed in 1 eye, and it resolved spontaneously without complication. In 4 eyes with ripcord retraction, the ripcord was removed with forceps after a small conjunctival incision in 2 eyes with elevated IOP. Intentional ripcord removal was performed in 43 (72.9%) eyes. There were no ripcord-related complications, including conjunctival erosion, dellen formation, infection, or filament formation.

The ripcord was removed at a mean postoperative day of 57.4 ± 45.3, with a mean IOP of 19.8 ± 8.2 mmHg. Following ripcord removal, IOP decreased to 11.9 ± 6.2 mmHg immediately (30 min), 12.4 ± 5.2 mmHg one week later, and 14.1 ± 7.6 mmHg one month later (Table 3). No significant differences were observed between the two groups for these variables (*p* > 0.05).

There were no bleb-related complications, including wound leakage, infection, avascular bleb formation, device exposure, tube occlusion, or device dislocation.

## 4. Discussion

In the present study, we evaluated the surgical outcomes of A-stream implantation using various procedural techniques, with particular emphasis on device coverage and ripcord placement. To date, this is the only report presenting various surgical options for A-stream implantation. This study highlights that exploring diverse surgical approaches can improve the accessibility of A-stream implantation by accommodating individual surgeons’ preferences.

Compared to the results of a previous study [1], our data demonstrated similar outcomes: a success rate of 93.9% vs. 86.4%, a mean IOP reduction from 26.9 to 11.9 mmHg vs. 26.1 to 14.5 mmHg, and further IOP reduction after ripcord removal from 18.3 to 11.5 mmHg vs. 19.8 to 14.4 mmHg. The slightly lower success rate and reduction in IOP observed in the present study may be attributable to differences in patient characteristics.

Adequate coverage of GDD is essential for preventing device exposure. In this study, we evaluated several techniques for GDD coverage, including scleral flap formation, conjunctival coverage alone, pericardial patch grafting, and MPTAF. During the 6-month postoperative follow-up period, no cases of device exposure were observed, making it difficult to determine which method is superior in preventing exposure. Despite the absence of exposure in this cohort, conjunctival coverage alone may pose a risk when the overlying conjunctiva is thin. Pericardial patch grafting, while effective, requires additional graft material and supplementary anchoring procedures. Scleral flap formation, though widely used in trabeculectomy, can be time-consuming—particularly for surgeons lacking experience with scleral flap-based techniques. Furthermore, inserting the tube beneath a scleral flap and securing the device by placing sutures anterior and posterior to the wing can be technically more challenging than direct scleral entry. In contrast, the MPTAF technique requires no additional material and is relatively easy to perform with a short learning curve.

**Figure 3:**
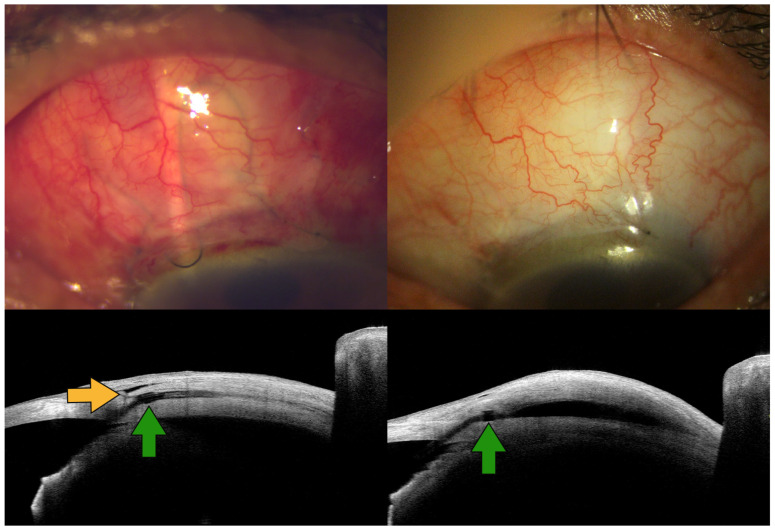
Appearance of the filtering bleb (**upper row**) and cross-sectional images obtained using anterior segment optical coherence tomography (**lower row**) on postoperative day 1 (**left column**) and day 56 (**right column**) following ripcord removal. The ripcord pathway, including anterior limbal loop, can be visualized under the bleb (**top left**). Green arrows indicate the wing of the A-stream. The anteriorly advanced mid-posterior Tenon’s capsule flap is anchored to the sclera (yellow arrow), providing substantial tissue coverage over the A-stream.

An additional possible benefit of the MPTAF technique could be the prevention of avascular bleb formation. Avascular blebs can result in ocular hypotony or infection due to aqueous leakage [9,10]. Therefore, preventing their formation is a critical consideration following glaucoma surgery. When the MPTAF technique is employed, the surgical site is covered with mid-posterior Tenon’s capsule, resulting in a thicker, more diffuse, and more posteriorly located bleb wall overlying the A-stream implant, as shown in Figure 3. This enhanced tissue coverage may reduce the risk of avascular, cystic, anterior bleb formation, thereby improving the long-term safety profile of the procedure. Previous studies have also reported that coverage of the scleral flap with Tenon’s capsule during trabeculectomy reduces the likelihood of developing avascular cystic blebs [4,5].

Despite the presence of the intraluminal ripcord, early postoperative hypotony was the most common complication following A-stream implantation [1]. Therefore, minimizing early postoperative hypotony remains a key concern in A-stream implantation. We believe that coverage of the aqueous drainage site with mid-posterior Tenon’s capsule may help reduce the risk of hypotony compared to techniques lacking this coverage. Chan et al. [5] also hypothesized that coverage of the scleral flap with Tenon’s capsule may help prevent overfiltration following trabeculectomy. However, it remains unclear whether the MPTAF technique offers an advantage over the scleral flap approach in terms of hypotony prevention.

It has been suggested that fibroblast proliferation within the Tenon’s capsule may induce scarring after trabeculectomy [11,12]. This raises the concern that the MPTAF technique might negatively affect surgical success. However, in the present study, eyes treated with MPTAF did not show lower success rates compared to those without MPTAF or to the previous study [1]. In addition, prior reports on Tenon’s capsule coverage during trabeculectomy similarly found that such coverage did not compromise surgical success [4,5].

For successful application of the MPTAF technique, several key issues merit attention. During the MPTAF technique, excessive advancement of the Tenon’s capsule should be avoided, as it may create stretching forces on the tissue and compromise the formation of an adequate bleb space for aqueous outflow. When Tenon’s capsule advancement is not readily achievable, meticulous dissection between the conjunctiva and Tenon’s capsule, as well as between the Tenon’s capsule and the underlying sclera, is recommended [6]. This approach mobilizes as much mid-posterior Tenon’s capsule as possible, allowing it to be sutured to the limbus without traction [6]. Another important consideration is the adequate application of an antimetabolite beneath the MPTAF, following a wide and thorough dissection of the anticipated bleb area to minimize postoperative fibrosis. Separate anchoring sutures to the scleral surface are also important to prevent slippage of the posterior Tenon’s capsule.

Stepwise postoperative flow control is a key feature of a tube equipped with an intraluminal ripcord [1,2,3]. A similar concept has been applied to recently introduced devices such as the Preserflo MicroShunt (Santen, Osaka, Japan) [13] and the Paul Glaucoma Implant (Advanced Ophthalmic Innovations, Singapore) [14]. However, inserting a thread into the small lumen of these tubes intraoperatively may require additional time and effort. In this regard, the pre-loaded intraluminal thread of the A-stream may offer a distinct advantage over other GDDs. To achieve a successful outcome, the ripcord must possess characteristics of stability, safety, and ease of removal. In terms of stability, our clinical experience with the first 25 cases revealed instances of ripcord displacement or retraction, possibly reflecting real-world challenges encountered by surgeons unfamiliar with the A-stream device. To prevent such dislocation, tight suturing of the ripcord to the scleral surface is recommended [1]. However, this method may offer insufficient friction for secure fixation. We propose that placing the ripcord beneath a sub-scleral pathway offers more contact surface area than suturing alone. Therefore, we adopted the anterior limbal loop technique, which incorporates two sub-scleral passes—before and after the loop segment. An alternative approach applies this concept posterior to the tube, creating a sub-scleral passage before placing the remainder of the ripcord on the conjunctival surface. A further theoretical advantage of anterior loop placement over posterior techniques lies in the reduced risk of bleb fibrosis. Although the ripcord’s diameter is relatively small, its prolonged presence across the bleb may induce fibrosis during wound healing.

Regarding safety, long-term placement of the ripcord (typically several months) must not provoke complications such as foreign body sensation, conjunctival erosion, dellen formation, infection, or filament formation. The likelihood of irritation may depend on the angle between the loop and ocular surface as well as the size of the loop, with smaller angles and loop sizes being better tolerated. A surgical tip for minimizing irritation involves creating a shallow needle track parallel to the ocular surface and keeping the distance between entry and exit points minimal. Another useful technique involves either burying the loop in a corneal groove or covering it during conjunctival suturing. Based on our clinical observation, the anterior loop has not caused significant discomfort or irritation-related complications. Loop removal was easily performed at the slit lamp by grasping the loop with forceps and pulling it out. In cases where the loop was buried beneath the conjunctiva, a needle was used to access and remove it.

A limitation of the anterior loop technique is that it may be difficult to perform if the partial removal of the ripcord is required. Posteriorly placed ripcords allow trimming of the remnant segment after partial removal. However, partially removing an anterior loop may lead to an enlarged, exposed loop, increasing the risk of discomfort or other complications. Therefore, when partial removal is anticipated, posterior placement of the ripcord may be preferable.

Corneal endothelial cell damage is a major concern for GDDs implanted in the anterior chamber. In the present study, no significant change in ECC was observed during the follow-up period, and no corneal complications were noted on clinical examination. To date, limited data exist regarding this issue, highlighting the need for further studies with longer follow-up periods.

The present study has several limitations, including a relatively small number of patients and a short follow-up period. Given that A-stream implantation is currently the primary surgical option in our clinic, and that the acquisition of clinical data—including anterior segment optical coherence tomography images of intrableb structures—is ongoing, we anticipate that more valuable insights will be presented in a subsequent report.

In conclusion, A-stream implantation, when combined with the MPTAF technique and limbal loop ripcord placement, appears to be a safe and effective surgical option, with reduced risk of device exposure and ripcord-related complications.

## Figures and Tables

**Table 1:** Clinical characteristics of eyes at baseline visit.

	Group A (*n* = 25)	Group B (*n* = 34)	*p* Value
Age (years)	61.0 ± 12.6	56.9 ± 12.9	0.221 *
Female: male (*n*)	7:18	8:26	0.462 ^†^
Diagnosis (*n*)			0.255 ^‡^
Primary open-angle glaucoma	16	23	
Neovascular glaucoma	0	1	
Pseudoexfoliation glaucoma	6	2	
Angle closure glaucoma	0	3	
Uveitic glaucoma	2	3	
Other secondary glaucoma	1	2	
Previous incisional glaucoma surgery (*n*)	7	5	0.177 ^†^
Pseudophakia (*n*)	16	17	0.305 ^†^
Visual acuity (logMAR)	0.28 ± 0.51	0.49 ± 0.84	0.281 *
Intraocular pressure (mmHg)	27.4 ± 9.8	25.1 ± 11.6	0.415 *
Number of medications (*n*)	2.7 ± 0.8	2.2 ± 0.7	0.011 *
Visual field test			
Mean deviation (dB)	−14.45 ± 8.66	−16.92 ± 8.43	0.354 *
Visual field index (%)	52.3 ± 28.9	50.1 ± 28.2	0.302 *
Corneal endothelial cell count (cells/mm^2^)	2044.5 ± 699.1	2259.1 ± 604.8	0.254 *

logMAR, logarithm of the minimum angle of resolution. Data are presented as mean ± standard deviation or number of eyes. * independent *t*-test, ^†^ Fisher’s exact test, ^‡^ chi-square test.

**Table 2:** Complications after A-stream implantation.

	Group A	Group B	*p* Value *
Numeric hypotony (<6 mmHg)			
Postoperative 1 week	11 (44.0%)	13 (38.2%)	0.429
Postoperative 1 month	2 (8%)	1 (3.7%)	0.471
Symptomatic hypotony (with complications)			
Postoperative 1 week			
Choroidal effusion	2 (8.0%)	5 (14.7%)	0.359
Shallow anterior chamber	0	0	
Hypotony maculopathy	0	0	
Postoperative 1 month	0	0	
Hyphema	1 (4.0%)	0	0.424
Ripcord-related events			0.071
Dislocation	3 (12.0%)	0	
Retraction into subconjunctival space	4 (16.0%)	0	

* Fisher’s exact test.

**Table 3:** Intraocular pressure (IOP) change after ripcord removal.

	Group A	Group B	*p* Value
Number of eyes	15 (60.0%)	28 (82.4%)	0.054 *
Duration between operation and ripcord removal (days)	46.5 ± 43.8 (range, 13–181)	63.3 ± 45.8 (range, 21–191)	0.250 ^†^
IOP immediate before removal (mmHg)	20.4 ± 10.3 (range, 11–46)	19.4 ± 7.0 (range, 13–43)	0.739 ^†^
IOP immediate after removal (mmHg)	9.5 ± 6.5 (range, 4–18)	12.4 ± 6.1 (range, 5–35)	0.406 ^†^
IOP 1 week after removal (mmHg)	12.7 ± 6.2 (range, 3–23)	12.3 ± 4.9 (range, 3–22)	0.872 ^†^
IOP 1 month after removal (mmHg)	15.2 ± 8.7 (range, 4–36)	13.6 ± 7.2 (range, 3–38)	0.579 ^†^

* Fisher’s exact test, ^†^ independent *t*-test.

## Data Availability

Upon reasonable request, the corresponding author will provide access to the datasets utilized or examined in this study.

## Supplementary Materials

The following supporting information can be downloaded at https://www.mdpi.com/article/10.3390/jcm15010056/s1, Video S1: surgical procedures of A-stream implantation.

## Abbreviations

The following abbreviations are used in this manuscript:

A-stream	A-stream glaucoma shunt
MPTAF	Mid-posterior Tenon’s capsule advancement flap
IOP	Intraocular pressure
GDD	Glaucoma drainage device
